# Comparison of amplification enzymes for Hepatitis C Virus quasispecies analysis

**DOI:** 10.1186/1743-422X-2-41

**Published:** 2005-04-22

**Authors:** Stephen J Polyak, Daniel G Sullivan, Michael A Austin, James Y Dai, Margaret C Shuhart, Karen L Lindsay, Herbert L Bonkovsky, Adrian M Di Bisceglie, William M Lee, Chihiro Morishima, David R Gretch, the HALT-C Trial Group

**Affiliations:** 1 Virology Division, Department of Laboratory Medicine, University of Washington, Seattle, WA, USA; 2 Department of Microbiology, University of Washington, Seattle, WA, USA; 3 Department of Pathobiology, University of Washington, Seattle, WA, USA; 4 Department of Biostatistics, University of Washington, Seattle, WA, USA; 5 Department of Medicine, University of Washington, Seattle, WA, USA; 6 Department of Pediatrics, University of Washington, Seattle, WA, USA; 7 Division of Gastrointestinal and Liver Diseases, University of Southern California, Los Angeles, CA, USA; 8 Liver-Biliary-Pancreatic Center and the General Clinical Research Center, University of Connecticut Health Center, Farmington, CT, USA; 9 Division of Gastroenterology and Hepatology, Saint Louis University School of Medicine, St. Louis, MO, USA; 10 Division of Digestive and Liver Diseases, University of Texas Southwestern Medical Center, Dallas, TX, USA

**Keywords:** hepatitis C virus, HALT-C, quasispecies, hypervariable region, E2

## Abstract

**Background:**

Hepatitis C virus (HCV) circulates as quasispecies (QS), whose evolution is associated with pathogenesis. Previous studies have suggested that the use of thermostable polymerases without proofreading function may contribute to inaccurate assessment of HCV QS. In this report, we compared non-proofreading (Taq) with proofreading (Advantage High Fidelity-2; HF-2) polymerases in the sensitivity, robustness, and HCV QS diversity and complexity in the second envelope glycoprotein gene hypervariable region 1 (E2-HVR1) on baseline specimens from 20 patients in the HALT-C trial and in a small cohort of 12 HCV/HIV co-infected patients. QS diversity and complexity were quantified using heteroduplex mobility assays (HMA).

**Results:**

The sensitivities of both enzymes were comparable at 50 IU/ml, although HF-2 was more robust and slightly more sensitive than Taq. Both enzymes generated QS diversity and complexity scores that were correlated (r = 0.68; p < 0.0001, and r = 0.47; p < 0.01; Spearman's rank correlation). QS diversity was similar for both Taq and HF-2 enzymes, although there was a trend for higher diversity in samples amplified by Taq (p = 0.126). Taq amplified samples yielded complexity scores that were significantly higher than HF-2 samples (p = 0.033). HALT-C patients who were HCV positive or negative following 20 weeks of pegylated IFN plus ribavirin therapy had similar QS diversity scores for Taq and HF-2 samples, and there was a trend for higher complexity scores from Taq as compared with HF-2 samples. Among patients with HCV and HIV co-infection, HAART increased HCV QS diversity and complexity as compared with patients not receiving therapy, suggesting that immune reconstitution drives HCV QS evolution. However, diversity and complexity scores were similar for both HF-2 and Taq amplified specimens.

**Conclusion:**

The data suggest that while Taq may overestimate HCV QS complexity, its use does not significantly affect results in cohort-based studies of HCV QS analyzed by HMA. However, the use of proofreading enzymes such as HF-2 is recommended for more accurate characterization of HCV QS in vivo.

## Background

HCV exists as quasispecies (QS) in infected individuals, consisting of a predominant viral variant and related, yet genetically distinct minor variants [1]. The study of HCV QS has historically focused on the hypervariable region 1 (HVR1) of the second envelope (E2) glycoprotein gene [2,3], the most variable region of the HCV genome. Early studies revealed that E2-HVR1 is a target of neutralizing antibodies [4-10]. Immune pressure is thought to be chiefly responsible for the fixation of the mutations in this region of the E2 gene.

HCV QS have been analyzed in many different patient cohorts. HVR1 QS evolution may reflect progression of liver disease [11-15]. HCV QS also reflect the outcome of acute HCV infection [16], and responses to antiviral therapy [17]. More recent studies have investigated the effect of HCV/HIV co-infection on HCV QS dynamics [18,19]. In the current study, we analyzed 20 baseline samples from the HALT-C trial and 12 HCV-HIV co-infected patient samples. The HALT-C study is a randomized multi-center clinical trial to assess the effects of long-term pegylated interferon-α (peg-IFN) therapy on the progression of liver fibrosis and development of decompensated liver disease in hepatitis C patients who are non-responders to prior pegylated IFN plus ribavirin therapy [20,21].

Viral QS have been analyzed by many techniques. Cloning and sequencing is the gold standard. Electrophoretic mobility-based assays, including single strand conformation polymorphism analysis (SSCP) and heteroduplex mobility analysis (HMA) allow determination of HCV QS heterogeneity without the need for sequencing (reviewed in [22]). HMA was originally described for analyzing the sequence heterogeneity of the envelope gene of human immunodeficiency virus (HIV) [23,24]. HMA involves hybridization of a radioactive probe generated from a QS variant to either heterogeneous PCR reaction products derived by direct PCR amplification from clinical specimens, or to homogeneous HVR1 sequences derived from cloned QS variants (clonal frequency analysis, CFA; [25]). Hybridizations between the probe and various target sequences result in the formation of double stranded DNA molecules (heteroduplexes) that produce shifts when the hybrids are separated on non-denaturing polyacrylamide gels. The shifts are determined by comparison to a homoduplex probe control (probe hybridized to itself). The extent of the heteroduplex shift compared to the homoduplex control is proportional to the degree of sequence divergence between the two DNA molecules. We have shown an excellent correlation between genetic diversity and complexity (number of variants) of individual QS variants derived by HMA as compared with standard cloning and sequencing of the HVR1 [11-13,25,26]. Moreover, HMA can be applied to studies of HCV QS evolution, in the context of therapy, transmission, and pathogenesis [11,12,25-29].

Taq polymerase is the enzyme used in most studies of HCV QS analysis. However, given that Taq lacks proofreading activity, mathematical debates have been raised to suggest that QS evolution is overestimated by Taq induced mutations [30]. Indeed, in one report, use of Taq increased the proportion of minor QS variants [31], and we have found that, in general, QS diversity is lower if proof-reading enzymes are used instead of Taq [32]. But the question arises as to whether Taq induced mutations affect the outcomes/conclusions in cohort-based studies on HCV QS. Thus, in the current study, we compared Taq and HF-2 polymerases in terms of sensitivity and robustness, and in terms of evaluating HCV QS diversity and complexity as assessed by CFA of the E2-HVR1 on baseline specimens from 20 patients in the HALT-C trial and 12 HCV-HIV co-infected patients receiving or not receiving HAART.

## Results

Clinical characteristics of the HALT-C and HCV-HIV co-infected patients are depicted in Tables 1 and 2. For HALT-C patients, all patients were infected with HCV genotype 1, with 10/20 (50%) being genotype 1a, and 7/20 (35%) being genotype 1b. Subtype designations were not obtained for 2/20 (10%) genotype 1 patients, and 1 patient was designated as type 1a or 1b. 16/20 (80%) patients were male. The average age of patients was 48.9 years, and average viral load was 10^6.56 ^IU/ml (3.6 × 10^6 ^IU/ml). For the HCV/HIV co-infected patients, all patients were infected with HCV genotype 1, with 10/12 (83%) being genotype 1a, and 2/12 (17%) being genotype 1b. Eight of 12 patients (67%) were male. The average age of the co-infected patients was 44 years, and average viral load was 10^6.53 ^IU/ml (3.3 × 10^6 ^IU/ml).

**Table 1:** Clinical and virological characteristics of the 20 HALT-C patients.

Patient	Age	Gender	Genotype	Serum HCV RNA at W00 (log_10_)	Diversity		Complexity	
					Taq	HF-2	Taq	HF-2

1	52	M	1a	6.55	0.9953	1.0000	7	1
2	54	F	1b	6.49	0.9905	1.0000	5	1
3	51	M	1a/b	6.99	0.9958	1.0000	5	1
4	42	M	1a	6.81	0.9877	0.9894	6	2
5	56	M	1a	7.13	0.9979	1.0000	4	1
6	42	M	1	6.55	0.9903	0.9912	8	6
7	73	M	1b	6.78	0.9892	0.9877	7	5
8	45	M	1	6.37	0.9193	0.9805	7	6
9	53	M	1a	7.06	0.9954	1.0000	2	1
10	47	M	1b	6.35	0.9845	0.9994	4	3
11	19	M	1b	5.72	0.9964	0.9994	2	2
12	49	M	1a	7.06	0.9979	0.9986	3	3
13	42	F	1b	6.26	0.9993	0.9781	2	2
14	42	M	1a	7.30	0.9810	0.9685	8	9
15	61	M	1b	5.85	0.9858	0.9624	4	5
16	54	M	1b	6.93	0.9047	0.9409	6	9
17	45	F	1a	6.72	0.9962	0.9917	2	5
18	47	F	1a	6.14	0.9966	0.9984	4	2
19	50	M	1a	6.37	0.9962	0.9968	4	3
20	53	M	1a	5.81	0.989	0.9943	6	5
**AVG**	**48.85**			**6.56**	**0.9845**	**0.9889**	**4.8**	**3.6**

HCV genotypes were determined by INNO-LiPA. HCV RNA was quantified with the Roche COBAS Monitor assay, and is expressed as log_10 _IU/mL. QS heterogeneity was assessed using the clonal frequency analysis (CFA) technique. Diversity scores represent the average heteroduplex mobility ratios (HMR) for either Taq or HF-2 enzymes. Complexity scores represent the total number of distinct gel shift variants analyzed by CFA. W00 represents the week 0 or baseline sample. AVG represents average.

**Table 2:** Clinical and virological characteristics of the 12 HCV/HIV co-infected patients.

Patient	Age	Gender	Genotype	Serum HCV RNA (log_10_)	Diversity		Complexity	
					Taq	HF-2	Taq	HF-2

1	49	F	1a	6.37	0.9856	0.9616	4	5
2	40	M	1a	5.96	0.9987	0.9978	2	2
3	36	F	1b	5.53	1.0000	1.0000	1	1
4	38	M	1a	6.75	0.9313	0.9715	8	11
5	42	M	1a	6.94	1.0000	1.0000	1	1
6	40	M	1b	5.55	0.9912	0.9997	5	2
7	41	M	1a	6.39	0.9909	1.0000	6	1
8	45	F	1a	6.25	0.9856	0.9468	4	7
9	47	M	1a	5.68	0.9945	0.9996	2	2
10	41	M	1a	5.92	0.9575	0.9430	10	10
11	61	F	1a	6.83	0.9389	0.9283	7	6
12	51	M	1a	6.99	0.9890	0.9638	2	7
**AVG**	**44.2**			**6.53**	**0.9803**	**0.9760**	**4.33**	**5.0**

HCV genotypes were determined by INNO-LiPA, while HCV RNA was quantified with the Roche COBAS Monitor assay, and is expressed a log_10_IU/mL. QS heterogeneity was assessed using the clonal frequency analysis (CFA) technique. Diversity scores represent the average heteroduplex mobility ratios (HMR) for either Taq or HF-2 enzymes. Complexity scores represent the total number of distinct gel shift variants analyzed by CFA. AVG represents average.

**Figure 1:**
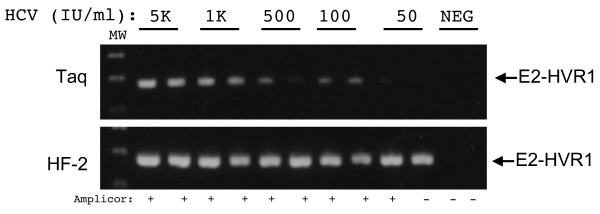
Comparison of the sensitivity of the E2-HVR1 PCR using Taq and HF-2 enzymes. RNA was extracted from duplicate serial dilutions of a WHO HCV standard and RT-PCR was performed with Taq and HF-2 enzymes. The dilutions corresponded to 50,000 (5K), 1,000 (1K), 500, 100, and 50 IU/ml, and are indicated above each lane. The position of the 176 bp E2-HVR1 is indicated with arrows. MW represents the 100 base pair DNA molecular weight marker. Below each lane is the result of testing of the same dilution of the standard with the Roche COBAS Amplicor assay. The result of this test gives a positive (+) or negative (-) result.

Figure 1 presents the sensitivity of the E2-HVR1 PCR using Taq versus HF-2 polymerases. Serial dilutions of the HCV international standard were run through the assay, and PCR products were visualized on agarose gels. As shown in Fig. 1, Taq produced amplification products at all dilutions, with 1 of 2 duplicate samples giving a signal at a dilution of 50 IU/ml. However, the HF-2 enzyme produced more PCR product than Taq at all dilutions and both replicates were positive at 50 IU/ml. Below each lane is the result of Roche COBAS Amplicor qualitative RT-PCR testing of the same serum specimens, which are scored as positive (+) or negative (-). At 50 IU/ml the Amplicor test was positive for 1 of the 2 duplicates. The data indicate that the sensitivities of the Taq and HF-2 enzymes were similar to the Amplicor assay, and the HF-2 enzyme was slightly more robust and sensitive than Taq polymerase.

**Figure 2:**
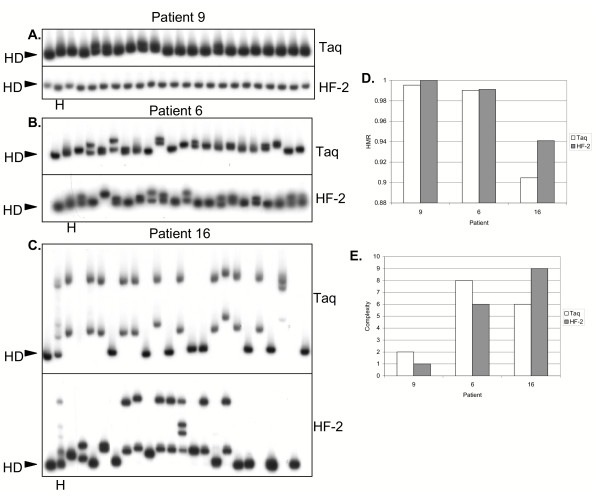
Representative autoradiograms of clonal frequency analyses of HALT-C patients with low (patient 9, panel A), intermediate (patient 6, panel B) and high (patient 16, panel C) QS diversity and complexity. E2-HVR1 RT-PCRs were performed with Taq and HF-2 enzymes. PCR products were cloned as described in the Materials and Methods, and individual colonies were picked and re-amplified. Lane 1 represents the homoduplex (HD) control and represents the probe hybridized to itself. Lane 2 represents the heteroduplex profile of the heterogenous (ie not cloned) E2-HVR1 PCR product and is designated "H". Panels D and E are graphical summaries of HMR and Complexity in the 3 patients.

The 2 enzymes produced QS diversity and complexity scores that were correlated (Linear regression analysis: R-squared: 0.4113, p < 0.0001 for diversity, and R-squared: 0.311, p < 0.001 for complexity; Spearman's rank correlation test: r= 0.68, p < 0.0001 for diversity, and r = 0.47, p < 0.01 for complexity). Figure 2 depicts representative clonal frequency analyses (CFA) of HALT-C baseline samples from patient 9 (figure 2A), patient 6 (figure 2B) and patient 16 (figure 2C), amplified by both Taq and HF-2 enzymes. The first lane of each gel represents the homoduplex probe control, obtained by hybridizing the radiolabeled probe to its non-labeled self. The homoduplex (designated as "HD" in the figure) serves as the reference point for all comparisons of individual clonal gel shift patterns. The second lane of each gel represents the heterogenous (ie non-clonal) PCR product, which contains all the QS variants amplified from the serum sample, and is designated as "H" in the figure. For each CFA, a shift control is also included, which involves the hybridization of the probe hybridized to a different HVR1 PCR product. In all cases, shift controls produced clearly identifiable shifts, indicating the hybridization reaction and electrophoresis was successful (data not shown). An internal control is also derived from a comparison of the heterogenous PCR product ("H") versus CFA gel shift patterns. If the gel shift patterns of the individual clones are representative of the heterogenous PCR product, the CFA was successful [11,26,27].

As shown in Figure 2A, patient 9 had very few discernible gel shifts, and the shifts themselves were not very distinct from the homoduplex probe control. The same pattern was observed when either Taq or HF-2 was used. Indeed, HMR scores for Taq and HF-2 (0.9954 vs. 1.0000) were similar, as were the complexity scores for samples analyzed by Taq and HF-2 (2 vs.1 variants). The flat line nature of the CFA pattern was not due to a failure of the hybridization reaction, because the shift control (the same probe hybridized to a different HVR1 PCR product) produced a significant gel shift (data not shown). Moreover, the same pattern was observed when the heterogenous PCR product (prior to cloning) was hybridized to the probe (Figure 2A, lane 2). The results indicate that this patient had minimal QS heterogeneity. In contrast, patient 6 had clearly identifiable gel shifts when compared to the probe (Figure 2B). The same pattern was observed whether the reaction was performed with Taq or HF-2 enzyme. Again, Taq and HF-2 scores were similar both for HMR (0.9903 vs 0.9912 (Taq vs. HF-2)) and complexity (8 vs. 6 variants (Taq vs. HF-2)). The gel shift pattern observed with CFA was similar to the pattern observed when heterogenous PCR product from the same time point was hybridized to the probe (Figure 2B, lane 2). Patient 16 displayed even more marked QS heterogeneity (Figure 2C). This was quantitated both in terms of HMR (0.9047 vs. 0.9409 (Taq vs. HF-2)) and complexity (6 vs. 9 variants, (Taq vs. HF-2)). The bar graphs in Figures 2D and 2E summarize the diversity (HMR) and complexity data, and confirm the progressively increasing QS diversity and complexity in patients 9, 6, and 16. Note that increased QS diversity is reflected as a decrease in HMR.

Furthermore, Taq gave lower HMRs indicative of higher diversity in all 3 patients, and higher complexity scores in 2 of the 3 patients. The data suggest that Taq may overestimate HCV QS genetic diversity and complexity when analyzed by HMA.

**Figure 3:**
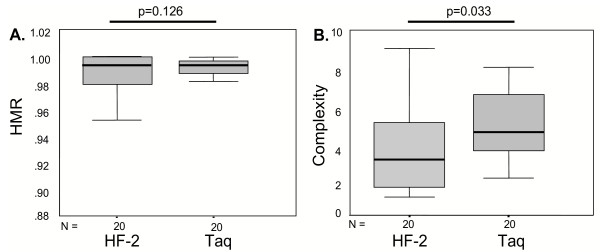
Comparison of quasipecies genetic diversity (assessed by HMR, Panel A) and complexity (panel B) values in samples processed with Taq or HF-2 polymerases in HALT-C baseline specimens. The box plots represent the means and ranges of the HMR and complexity scores for 400 HVR1 clones amplified by each polymerase, for a total of 800 clones (20 patients × 20 clones/patient × 2 enzymes). Error bars represent standard deviations. Significance values above each panel were derived from Wilcoxon Signed Ranks tests.

To further examine this issue, CFA was performed for 20 HALT-C baseline specimens, and QS diversity scores and complexity scores were determined. These data are summarized in Table 1 and depicted graphically in Figure 3. Figure 3A depicts the HMR results, while Figure 3B depicts the complexity scores for the 20 HALT-C patients analyzed by both enzymes. The average HMR for Taq amplified samples was 0.9845 (range 0.9047–0.9993), while the average HMR for the HF-2 enzyme was 0.9889 (range 0.9407–1.000). Taq samples appeared somewhat more diverse than HF-2 samples. However, this trend did not reach statistical significance (p = 0.126). Similarly, the average complexity for Taq amplified samples was 4.8 (range 2–8), while the average complexity for the HF-2 enzyme was 3.6 (range 1–9). QS complexity scores were higher in samples amplified by Taq as compared with samples amplified by HF-2 (p = 0.033).

Cumulatively, the data indicate that Taq overestimates HCV QS genetic diversity and complexity. However, what is not clear is whether this tendency for overestimation by Taq impacts QS scores in clinical studies.

**Figure 4:**
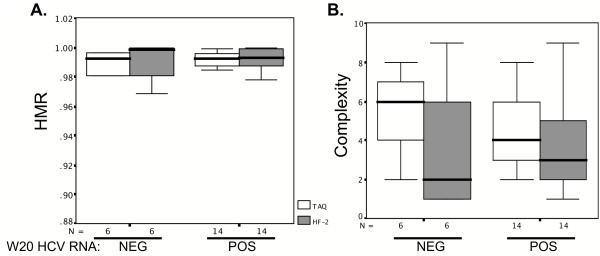
Comparison of quasipecies genetic diversity (assessed by HMR, Panel A) and complexity (panel B) scores generated with Taq or HF-2 polymerases, in 20 HALT-C samples who were HCV RNA negative (N = 6) or HCV RNA positive (N = 14) at week 20 (W20) of pegylated IFN plus ribavirin therapy. The box plots represent the means and ranges of the HMR and complexity scores for 400 HVR1 clones amplified by each polymerase, for a total of 800 clones (20 patients × 20 clones/patient × 2 enzymes). Error bars represent standard deviations. Wilcoxon Signed Ranks tests determined that the differences between enzymes and patient groups were not statistically significant.

To address this issue, we grouped the 20 HALT-C patients according to whether they were serum positive or negative for HCV RNA following 20 weeks of pegylated IFN plus ribavirin therapy. By this criterion, 6 patients were HCV RNA negative and 14 patients were HCV RNA positive at week 20. Figure 4 presents the HMR and complexity scores for the 2 groups of patients and demonstrates that HMR and complexity scores were similar among patients who were HCV RNA negative or positive following 20 weeks of peg-IFN plus ribavirin, regardless of the enzyme used. There was a trend for HF-2 samples generating lower complexity scores as compared to Taq samples, but the difference was not significant. Note also that the lower complexity scores from HF-2 as compared with Taq processed samples among week 20 responders and non-responders mirrored the results when all patients were considered together (Figure 3).

**Figure 5:**
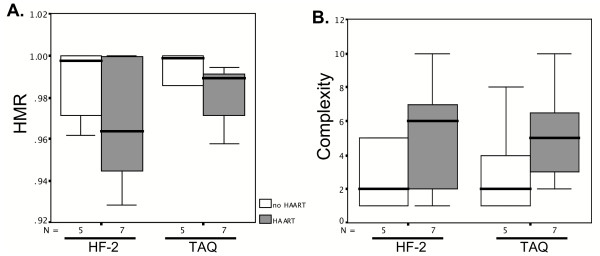
Comparison of quasipecies genetic diversity (assessed by HMR, Panel A) and complexity (panel B) values processed with Taq or HF-2 polymerases, in 12 HCV/HIV co-infected samples treated (N = 7) or not treated (N = 5) with HAART. The box plots represent the means and ranges of the HMR and complexity scores for 240 HVR1 clones amplified by each polymerase, for a total of 480 clones (12 patients × 20 clones/patient × 2 enzymes). Error bars represent standard deviations. Wilcoxon Signed Ranks tests determined that all differences were not statistically significant.

To further determine if the choice of amplification enzyme affects results in patient cohort studies, we compared QS diversity and complexity on samples from 12 HCV/HIV co-infected patients treated or not treated with highly active anti-retroviral therapy (HAART). As shown in Figure 5, patients receiving HAART had increased QS diversity (shown as a decrease in HMR in panel A) and complexity (panel B) as compared to patients who did not receive HAART. This trend was apparent regardless of whether Taq and HF-2 were used. The increase in diversity and complexity scores upon HAART did not reach statistical significance. Moreover, HMR and complexity scores were not significantly different between Taq and HF-2 samples. In fact, HAART-samples amplified by HF-2 showed a trend for higher complexity scores (complexity = 4) as compared with Taq samples (complexity = 3.2). These data suggest that although Taq may overestimate QS complexity, it likely does not mask potentially important clinical associations when HCV QS are analyzed by HMA.

## Discussion

In the current investigation, we found that the sensitivity of Taq and HF-2 enzymes in amplifying the E2-HVR1 were similar to the qualitative Roche COBAS Amplicor RT-PCR assay. The HF-2 enzyme generated more PCR product and was more sensitive than Taq. For HALT-C samples, QS diversity, expressed as an HMR, was similar for both Taq and HF-2 enzymes, although Taq tended to give higher diversity scores. HCV QS complexity was significantly higher in samples amplified by Taq as compared with samples amplified by HF-2. In contrast, in HCV/HIV co-infected samples, diversity and complexity scores were similar for both enzymes. The data from this limited cohort suggest that QS results are not significantly influenced by choice of polymerase when using the CFA method.

Our data indicate that Taq induced errors provide inflated estimates of HCV QS diversity and complexity. The data are in accord with previous mathematical models of HCV QS mutations [30], and a recent study that demonstrated that Taq induced errors can generate minor QS variants [31]. However, our results showed only a trend for increased HMR for Taq amplified HALT-C samples as well as for HMR and complexity scores for HCV/HIV co-infected samples. Based on the current results, it would seem that Taq and proof-reading enzymes are acceptable for HCV QS analyzed by CFA. However, further studies on larger patient cohorts need to be performed.

Genetic evolution in the E2-HVR1 is believed to reflect selective forces imposed on this domain by neutralizing antibodies [4-10]. It is also possible that cell mediated immune responses may impart selective pressures on HVR1. In the face of immune pressure, the plasticity of the HVR1 may allow the virus to persist. However, recent studies suggest that the HVR1, although it is the most variable region in the HCV genome, has certain constraints in its structure. It is intriguing that key basic residues are highly conserved, which maintains the chemicophysical properties and conformation of the HVR1 [33]. Because HVR1 is an exposed domain on the E2 protein, these data suggest that the positively charged HVR1 is involved in interactions with negatively charged molecules such as lipids, proteins, or glycosaminoglycans (GAGs). As such, the HVR1 may interact with GAGs facilitating host cell recognition and attachment [33]. In this regard, it has recently been shown that high pretreatment HCV QS diversity and complexity at baseline are associated with non-response to pegylated IFN ribavirin in HALT-C patients [34].

HAART therapy appeared to increase HCV QS diversity and complexity, regardless of the enzyme used, but this did not reach statistical signficance in this small cohort. These data are consistent with recent reports that suggest that HAART-induced immune reconstitution drives HCV QS evolution [18,19]. Additional prospective trials on larger patient cohorts are required to further examine the relationships between immune pressure, HCV QS evolution, and liver disease progression in patients co-infected with HIV and HCV.

## Materials and methods

### Patients

Twenty patients who met entry criteria for the HALT-C trial were included in the current study. Serum samples from baseline (week 0; (W00)), prior to the start of IFN therapy, were analyzed. Written, informed consent was provided by all patients, following institutional and trial specified IRB regulations. The design and conduct of the HALT-C trial have been described [21]. Briefly, in the initial or lead-in course of therapy, all patients are treated with pegylated interferon (Pegasys, Roche) plus ribavirin for a period of 24 weeks, with virologic assessment at 20 weeks to assess whether or not they are virological responders. Patients who are responders at week 20 continue treatment and receive a full course of 48 weeks of therapy. Those who remain HCV-positive in serum at 20 weeks are randomized to receive either continued low-dose pegylated interferon (without further ribavirin) or no further treatment beyond careful observation for the ensuing 3.5 years. Immunology and virology ancillary studies including QS analysis are aimed at increasing our understanding of the complex interactions of virus and host in this debilitating and widespread disease [34].

Samples from twelve patients co-infected with HCV and HIV were also analyzed. Seven of these patients received HAART, while 5 patients did not. All 32 patients were infected with HCV of genotype 1.

### E2-HVR1 RT-PCR Amplification and Cloning

HCV RNA was extracted from 100 μL of patient sera using HCV RNA isolation columns (Qiagen). RNA was resuspended in DEPC-treated water, and converted into cDNA using oligonucleotide primers and AMV reverse transcriptase, as described previously [26]. The same cDNA sample was then amplified using either Taq (Perkin Elmer, Wellesley, MA) or Advantage High Fidelity 2 (HF-2) (Clontech; Mountain View, CA) polymerases, using a nested PCR reaction as described [26]. To provide a hot start, AmpliWax (Perkin Elmer) beads were used to separate Taq enzyme and primers, whereas the HF-2 enzyme mixture contains an anti-Taq antibody. The primers generated the expected second round product of 176 base pairs. PCR products were excised and purified with the QiaEx purification system (Qiagen, Valencia, CA), ligated into pCR2 vector, and transformed into TOP10 cells as described [11].

### Sensitivity of E2-HVR1 PCR Assay

To determine the sensitivity of the E2-HVR1 PCR assay using Taq versus HF-2 polymerases, serial dilutions of an HCV World Health Organization (WHO) international standard [35] were prepared from 50,000 to 50 International Units/milliliter (IU/ml). RNA was extracted, converted into cDNA and amplified by nested PCR as described above. PCR products were separated on agarose gels. Aliquots of WHO standards were also run through the Roche Amplicor assay for internal comparison.

### HCV RNA quantitation and genotyping

HCV RNA was qualitatively measured by RT-PCR using the Roche COBAS Amplicor HCV test version 2.0 (RocheMolecular Systems, Branchburg, NJ), and quantitatively using Roche COBAS Amplicor HCV Monitor v. 2.0 (Roche Molecular Systems, Branchburg, NJ). Genotyping was performed using the INNO-LiPA HCV II Kit (Bayer Diagnostics, Emeryville, CA) All assays were performed according to manufacturer's specifications.

### Clonal Frequency Analysis (CFA)

For each cloned HVR1 PCR product, 20 colonies were picked directly into tubes for re-amplification of the second round PCR product. Thus, for each patient, 20 PCR products representing 20 individual QS clones derived from the baseline serum were analyzed. PCR products were visualized on ethidium bromide-stained agarose gels, and one HVR1 PCR product was randomly selected, purified as described above, and end-labeled with ^32^P ATP and T4 polynucleotide kinase. Unincorporated label was separated with Centrisep columns (Princeton Separations, Adelphia, NJ), and the labeled DNA probe was eluted from the column. Labeled probe was hybridized directly to each amplified PCR product for 2 hours at 55°C as described [11]. Hybrids were separated on 6% non-denaturing polyacrylamide MDE gels (Cambrex, Baltimore, MD) and visualized by autoradiography as described [11].

### Data Analysis

QS complexity was determined by counting the total number of unique gel shift patterns. QS genetic diversity was determined by deriving the average heteroduplex mobility of all clones relative to the homoduplex probe control. A heteroduplex mobility ratio (HMR) was calculated by dividing the distance in millimeters (mm) from the origin of the gel to the heteroduplex by the distance in mm from the origin to the homoduplex control. In cases where both strands of the heteroduplex were clearly distinguishable, the average of the distance of each strand of the heteroduplex was used to calculate heteroduplex mobility [25]. The HMRs for all variants in the population were averaged to provide the final HMR value. Non-parametric Wilcoxon Signed Ranks tests were used to compare the differences in QS complexity and diversity scores between the different enzymes. Linear regression analysis and Spearman's rank correlation tests were also used to determine the correlation of Taq and Ad-HF2 measurements of QS diversity and complexity.
